# Factors Related to Workplace Well-Being: Occupational Health and Safety of Older Workers in Hong Kong

**DOI:** 10.1093/geroni/igaa057.134

**Published:** 2020-12-16

**Authors:** Vincent Lee, Daniel W L Lai, Kin Cheung, Calvin Luk, Xue Bai, Jessica J Li, Joan Wu

**Affiliations:** The Hong Kong Polytechnic University, Kowloon, China

## Abstract

As Hong Kong is undergoing rapid population ageing over the past few decades, and this trend is expected to persist due to low fertility rates and extended life expectancy. This trend has significant implications for Hong Kong’s labor market and workforce. This study aims to gain a better understanding of the experiences, needs, and definitions of older workers in Hong Kong. It helps to draw attention to issues of occupational safety and health (OSH) risks and help identifying and promoting strategies and interventions for effectively supporting the ageing workforce in the workplace. The major objective of this research is to identify the OSH problems and challenges perceived by the older workers, employers and other stakeholders. Both quantitative and qualitative research methods are applied. A qualitative study with focus groups and individual in-depth interviews have been conducted with a representative sample of 70 stakeholders, including employees, employers, representative of labour unions, policymakers, NGO representatives and researchers in the related disciplines. We obtained their views on age-related changes within the older workforce and how such changes would affect their work ability, the OSH issues facing the older workers in different industries, and the facilitators and barriers associated with remaining in or joining the labour force. Feasible intervention strategies for effectively supporting the ageing workforce in the workplace are also being identified.

